# Tumor cuproptosis and immune infiltration improve survival of patients with hepatocellular carcinoma with a high expression of ferredoxin 1

**DOI:** 10.3389/fonc.2023.1168769

**Published:** 2023-06-08

**Authors:** Yingyao Quan, Wei Li, Rongrong Yan, Jing Cheng, Heng Xu, Lin Chen

**Affiliations:** ^1^ Department of Anesthesiology, Maternal and Child Health Hospital of Hubei Province, Hubei, Wuhan, China; ^2^ The Second Department of General Surgery, Zhuhai People’s Hospital (Zhuhai Hospital Affiliated with Jinan University), Guangdong, Zhuhai, China

**Keywords:** hepatocellular carcinoma, ferredoxin 1, prognosis, cuproptosis, tumor immune microenvironment

## Abstract

**Background:**

Cuproptosis is a novel cell death pathway dependent on cellular copper ions and ferredoxin 1 (FDX1). Hepatocellular carcinoma (HCC) is derived from healthy liver as a central organ for copper metabolism. It remains no conclusive evidence whether cuproptosis is involved in survival improvement of patients with HCC.

**Method:**

A 365–liver hepatocellular carcinoma (LIHC) cohort with RNA sequencing data and paired clinical and survival information was obtained from the The Cancer Genome Atlas (TCGA) dataset. A retrospective cohort of 57 patients with HCC with stages I/II/III was collected by Zhuhai People’s Hospital from August 2016 to January 2022. Low- or high-FDX1 groups were divided according to the median value of FDX1 expression. Cibersort, single-sample gene set enrichment analysis, and multiplex immunohistochemistry analyzed immune infiltration in LIHC and HCC cohorts. Cell proliferation and migration of HCC tissues and hepatic cancer cell lines were evaluated using the Cell Counting Kit-8. Quantitative real-time PCR and RNA interference measured and downregulated FDX1 expression. Statistical analysis was conducted by R and GraphPad Prism software.

**Results:**

High FDX1 expression significantly enhanced survival of patients with LIHC from the TCGA dataset, which was also demonstrated through a retrospective cohort with 57 HCC cases. Immune infiltration was different between the low– and high–FDX1 expression groups. Natural killer cells, macrophages, and B cells were significantly enhanced, and PD-1 expression was low in the high-FDX1 tumor tissues. Meanwhile, we found that a high expression of FDX1 decreased cell viability in HCC samples. HepG2 cells with FDX1 expression are sensitive to Cu^2+^, and interference of FDX1 promoted proliferation and migration of tumor cells. The consistent results were also demonstrated in Hep3B cells.

**Conclusion:**

This study reveals that cuproptosis and tumor immune microenvironment were together involved in improvement of survival in patients with HCC with a high expression of FDX1.

## Introduction

Primary liver cancer is the sixth most commonly diagnosed cancer and the third leading cause of cancer death worldwide (1). In 2020, there were approximately 906,000 new incident cases of liver cancer globally and 830,000 deaths (1). Hepatocellular carcinoma (HCC) accounts for approximately 80% of primary liver cancer (1, 2). The incidence rate of liver cancer is the highest increase for all sites in the USA (3, 4). In China, HCC ranks second only to lung cancer in cancer-related mortality (5). Although incidence and mortality of liver cancer had decreasing trend, population growth and aging still caused a large number of new cases in China (4). The main risk factors for HCC include hepatitis B virus (HBV), hepatitis C virus (HCV), excessive alcohol consumption, obesity, type 2 diabetes, smoking, and Aspergillus intake (6), and the major risk factors of HCC in China is chronic HBV infection or/and aflatoxin exposure (1). The decreasing trend may be related to infection control of HBV/HCV—vaccination of these viruses in China (7).

Copper is an essential trace element for the human body, which is utilized by specific proteins to involve in many biological processes, such as oxidative phosphorylation, antioxidant defense, metabolism of iron ions, and protein synthesis (8). The liver is the major regulatory organ of copper homeostasis. Copper is absorbed by the liver and distributes to other organs through the circulatory system. HCC is derived from healthy liver and also has many functions of the liver. Thus, cell proliferation and migration of HCC is also subject to influence of copper regulation. Cuproptosis, as a novel cell death pathway dependent on cellular copper ions and ferredoxin 1 (FDX1), distinguishes from cell apoptosis, cell necrosis, and ferroptosis (9). Copper accumulation in the liver induced cell death in the Atp7b-deficient (Atp7b^−/−^) mouse model (9). High expression of the cuproptosis key factor, FDX1 protein, may also promote the cuproptosis pathway of hepatic tumor cells. There remains a lack of strong clinical demonstration whether FDX1 expression could predict the survival of HCC cases.

In this study, a 365-liver hepatocellular carcinoma (LIHC) cohort from the TCGA dataset and a retrospective HCC cohort from Zhuhai People’s Hospital were enrolled to explore effect of FDX1 expression on patient survival with HCC. We further explored reasons of FDX1-caused survival difference from the cuproptosis pathway and tumor immune microenvironment change using HCC samples and hepatic tumor cells.

## Materials and methods

### RNAseq dataset

Gene expression RNA sequencing (RNAseq) dataset (HTSeq-Counts and HTSeq-FPKM) and clinical information with 424 samples were obtained from Genomic Data Commons (GDC) TCGA Liver Cancer (LIHC) (14 datasets) (https://xenabrowser.net/datapages/). A 365-LIHC cohort with RNAseq dataset and paired clinical and survival information was obtained from the dataset. The clinical information included age, gender, risk factor, pathologic stage, and family cancer history (**Table 1**). Patients with two or more risk factors (alcohol consumption, hepatitis B, hepatitis C, and others) were regarded as complex risk factors. Cutoff value according to HTSeq-counts median value of FDX1 expression (counts = 1267) was regarded as low- or high-FDX1 groups, and counts of >1,267 were considered as the high FDX1 expression and the remaining as the low expression.

**Table 1:** Clinical characteristics of LIHC from TCGA dataset.

Factor	LIHC (n = 365)
Age	61 (16–90)
Gender	
Male	246 (67.4%)
Female	119 (32.6%)
Risk factor	
No-risk factors	91 (24.9%)
Alcohol consumption	67 (18.4%)
Hepatitis B	73 (20%)
Hepatitis C	32 (8.8%)
Complex	84 (23%)
NA	18 (4.9%)
Pathologic stage	
Stage I	170 (46.6%)
Stage II	84 (23%)
Stage III	83 (22.7%)
Stage IV	4 (1.1%)
NA	24 (6.6%)
Family cancer history	
Yes	112 (30.7%)
No	204 (55.9%)
NA	49 (13.4%)

NA, Not Applicable.

### Patients and clinical data

The present study enrolled 57 HCC cases by Zhuhai People’s Hospital with I/II/III stages from August 2016 to January 2022. The clinical information contained age, gender, risk factor, pathologic stage, and family cancer history (**Table 2**). This research was approved by the Ethics Committee of Zhuhai People’s Hospital, and treatment was performed by the Chinese Society of Clinical Oncology guidelines. Extended follow-up data were retrospectively assembled with medical ethics committee approval. All patients provided their written informed consent to participate in this research. FDX1 expression in the HCC cohort was detected using quantitative real-time PCR. The median value of the relative expression of FDX1 (relative value = 1.33) was considered as a cutoff value, and relative value > 1.33 was considered as the high expression of FDX1 and the remaining as the low expression.

**Table 2:** Clinical characteristics of HCC in a retrospective cohort.

Factor	HCC (n = 57)
Age	60 (36–77)
Gender	
Male	38 (66.7%)
Female	19 (33.3%)
Risk factor	
No-risk factors	10 (17.6%)
Alcohol consumption	22 (38.6%)
Hepatitis B	19 (33.3%)
NA	6 (10.5%)
Clinical stage	
Stage I	24 (42.1%)
Stage II	13 (22.8%)
Stage III	20 (35.1%%)
Family cancer history	
Yes	12 (21.1%)
No	37 (64.9%)
NA	8 (14%)

NA, Not Applicable.

### Immune infiltration analysis

Immune infiltration analysis was performed using Cibersort (10) and the single-sample gene set enrichment analysis (ssGSEA) (11) methods. Cibersort calculated the proportion of 22 immune cells, and ssGSEA calculated the extent of infiltration of 28 immune cell types in each case with LIHC.

### Immunohistochemistry

Biopsy tissue samples were processed into 5-μm-thick formalin-fixed paraffin-embedded (FFPE) sections, which were stained using the Opal Seven-color IHC Kit (NEL797B001KT; PerkinElmer, USA). CD8 (clone SP16; ZA-0508, Zsbio; 1:100), CD68 (clone KP1; ZM-0060, Zsbio; 1:100), CD19 (clone UMAB103; ZM0038, Zsbio; 1:100), CD56 (clone UMAB83; ZM0057, Zsbio; 1:100), CD68 (clone KP1; ZM0060, Zsbio; 1:100), and programmed cell death-1 (clone UMAB199; ZM0381, Zsbio; 1:100) antibodies marked cytotoxic T cells, B cells, natural killer (NK) cells, macrophages, and PD-1 protein, respectively. The FFPE sections were, in turn, incubated with primary and secondary antibodies, visualized using tyramide signal amplification, and treated under a microwave oven, followed by labeling with the next antibody. After five antibodies staining, cell nucleus was stained by diamidino-phenyl-indole (DAPI). The sections were scanned and analyzed using PerkinElmer Vectra (Vectra 3.0.5; PerkinElmer, MA, USA).

### Cell culture

Hepatocellular HepG2 and Hep3B cells were purchased from the Cell Bank of Type Culture Collection of the Chinese Academy of Sciences (Shanghai, China). HepG2 and Hep3B cells were cultured in Dulbecco’s modified Eagle’s medium (Gibco) with 10% fetal bovine serum (Gibco), streptomycin (100 μg/ml) and penicillin (100 μg/ml) in a 5% CO_2_ and 37°C humidified incubator.

### Cell proliferation and migration

Proliferation of HepG2 and Hep3B cells was detected using the Cell Counting Kit-8 (CCK-8; Dojindo, Japan). Cells were cultured in 96-well plates and treated with 0, 10, 100, 1000 nM Elesclomol-CuCl_2_ (ratio, 1:1; MCE) for 24 h. CCK-8 assay was performed, and absorption was detected at 490 nm using a microplate reader. Migration of cells was detected using a Transwell culture test. Cells were seeded and cultured in Transwell filters for 24 h. The cells of upper surface in Transwell filter were removed. The cells of lower surface in Transwell filter were stained with crystal violet staining and imaged by an optical microscope. Or the cells of lower surface were transferred to 96-well plates and measured by CCK-8 assay.

### Real-time PCR

Quantitative real-time PCR was performed as previously described (12). Total RNAs were extracted using TRIzol reagent (Invitrogen, USA) from tumor tissues and cells after different treatments, reverse-transcribed to cDNA using the First Strand cDNA Synthesis Kit (Roche, Germany). Quantitative real-time PCR was performed with the SYBR Green Real-Time PCR Master Mix (ThermoFisher, USA) using the StepOnePlus Real-Time PCR System (ThermoFisher, USA). The primer sequences contained FDX1 (forward strand, 5′-GCCATTCTCTCACGCAAGGAT-3′; reverse strand, 5′-ACGTGGTAATCTGTGGTGCTT-3′) and glyceraldehyde-3-phosphate dehydrogenase (GAPDH) (forward strand, 5′- TGCACCACCAACTGCTTAGG-3′; reverse strand, 5′- GGCATGGACTGTGGTCATGAG -3′).

### RNA interference

HepG2 and Hep3B cells were seeded and cultured in six-well plates and grown to about 80% confluence. The cells were transfected with small interfering RNA of ferredoxin 1 (siFDX1) or negative control interfering RNA (siControl) using Lipofectamine 3000 (Invitrogen, USA) according to the manufacturer’s instructions. After 24 h, FDX1 expression was detected in the RNA interfering (RNAi) HepG2 and Hep3B cells through real-time PCR tests. In addition, the cells were the subsequent tests. FDX1 sequences of RNA interference were forward strand 5′-CUAACAGACAGAUCACGGUTT-3′ and reverse strand 5′-ACCGUGAUCUGUCUGUUAGTT-3′.

### Statistical analysis

Statistical analysis of this research was conducted by R software (version 4.2.1) and GraphPad Prism software (version 8.4.2). All data came from TCGA dataset, and analysis was performed using the survminer package in R software. Immune infiltration analysis was performed using CIBERSORT and GSVA packages. Survival analysis was performed using Kaplan–Meier analysis (log-rank test). Clinical data comparison was based on the two-tailed Mann–Whitney U-test. Cell experiment analysis was based on the unpaired t-test. A p-value of <0.05 was considered significant.

## Results

### High expression of FDX1 enhances survival of patients with LIHC

**Figure 1:**
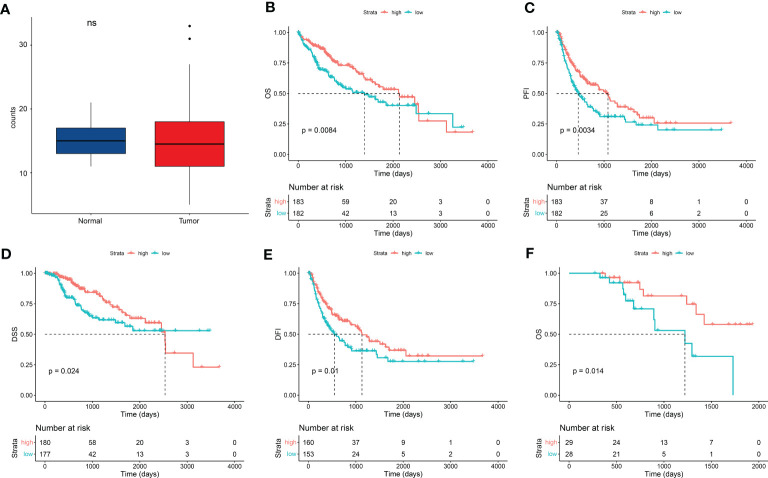
FDX1 expression improved patient survival in HCC. **(A)** FDX1 expression between normal and LIHC tissues from TCGA dataset (n = 50). Statistics based on the paired samples Wilcoxon signed-rank test. **(B–E)** Kaplan–Meier analysis of overall survival (OS) **(B)**, progression-free interval (PFI) **(C)**, disease-specific survival (DSS) **(D)**, and disease-free interval (DFI) **(E)** in liver hepatocellular carcinoma (LIHC) stratified by FDX1 expression (n = 365). Median value of FDX1 expression was determined as the cutoff point. All data came from TCGA dataset, and analysis was performed using the survminer package in R software. **(F)** Kaplan–Meier analysis of OS in an HCC patient cohort in the real world from Zhuhai People’s Hospital stratified by FDX1 expression (n = 57).

We obtained RNAseq dataset and clinical information of 424 samples from TCGA datasets and filtered 365 LIHC cases with RNAseq dataset and paired clinical and survival information. Clinical data of the LIHC cohort are shown in **Table 1**. This cohort mainly included LIHC cases of I–III stages [337 (92.3%)]. There were 256 (70.1%) patients with history LIHC risk factors, such as alcohol consumption, HBV, HCV, or/and complex risk factors, and 112 (30.7%) patients with family cancer history. First, we found that there was no significant different in expression of FDX1 between normal and LIHC tissues (**Figure 1A**). According to the count median value of FDX1 expression, the cohort was regarded as low and high groups of FDX1 expression. As shown in **Figures 1B–E**, overall survival (OS), progression-free interval (PFI), disease-specific survival (DSS), and disease-free interval (DFI) in the high-FDX1 group were significantly better than that in the low group. These results revealed that LIHC cases with a high FDX1 expression had a better survival.

To demonstrate the result, a retrospective HCC cohort from Zhuhai People’s Hospital was studied. This cohort contained 57 HCC cases with I/II/III stages from August 2016 to January 2022. There were 41 (72%) patients with history HCC risk factors and 12 (21%) patients with family cancer history (**Table 2**). FDX1 expression was detected in tumor samples of these cases using real-time PCR. Low and high groups were divided according to the median value of FDX1 expression. Five years of follow-up data indicated that OS was better in the high–FDX1 expression group than the low group (**Figure 1F**). The result further demonstrated that high expression of FDX1 was beneficial for survival and outcome of patients with HCC.

### Correlation between expression of FDX1 and LIHC clinical characteristic

**Figure 2:**
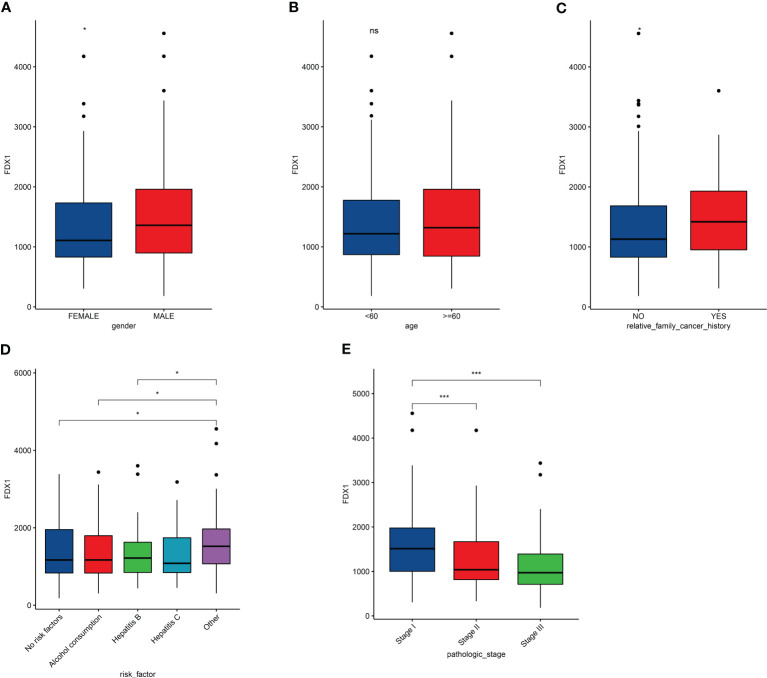
Effects of clinical characteristic on expression of FDX1 in LICH. **(A–E)** Correlation between FDX1 expression and gender **(A)**, age **(B)**, family cancer history **(C)**, risk factor **(D)**, and pathologic stage **(E)** in LIHC (n = 365). Statistics based on the two-tailed Mann–Whitney U-test. *p < 0.05, ***p < 0.001.

We further explored correlation between expression of FDX1 and LIHC clinical characteristic. In the LIHC cohort from the TCGA dataset, expression of FDX1 in male patients with LIHC was more than that in female patients (**Figure 2A**). In addition, expression of FDX1 was correlated with family cancer history (**Figure 2C**). There was no correlative among age in the LIHC cohort (**Figure 2B**). FDX1 expression in patients with LIHC with risk factors (alcohol consumption, hepatitis B, or hepatitis C) and in the no-risk factor group was no different, although there was a significant difference between patients with no-risk factor, alcohol consumption, or Hepatitis B and the complex group (**Figure 2D**). We further found that there was a significant correlation in the expression of FDX1 among pathologic stage (**Figure 2E**). In the LIHC cases with stage I, FDX1 expression was more than the stage II or stage III cases. In our retrospective LIHC cohort, there was no marked different between FDX1 expression and clinical information, but the variation trend of FDX1 in gender, family cancer history, and pathologic stage was similar with that in the LIHC cohort (**Figure S1**).

### FDX1 ameliorates tumor immune microenvironment of LIHC

**Figure 3:**
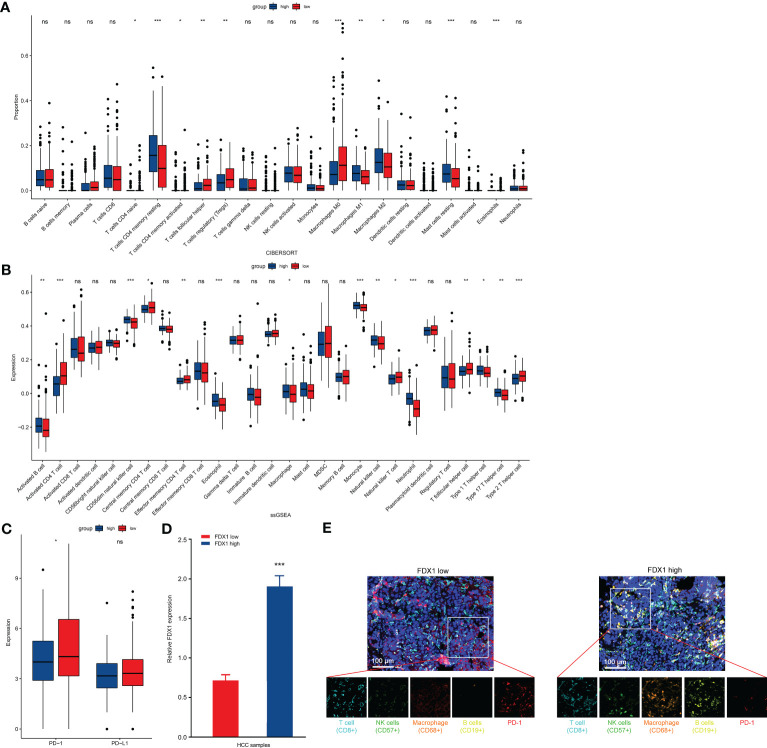
Comparison of immune characteristics between high– and low–FDX1 expression groups in HCC. **(A, B)** Proportion of immune cells **(A)** and expression of immune cells **(B)** between high and low groups of FDX1 expression in the LIHC cohort using cibersort analysis and ssGESA analysis, respectively. *p < 0.05, **p < 0.01, and ***p < 0.001; ns, no significance. **(C)** PD-1 and PD-L1 expression between the high– and low–FDX1 expression groups in the LIHC cohort. Statistics based on the two-tailed Mann–Whitney U-test. *p < 0.05; ns, no significance. **(D)** High/low expression of FDX1 in the HCC cohort (n = 5). Statistics based on the unpaired t-test. ***p < 0.001. **(E)** Representative mIHC images of LIHC samples stained with CD8+ T cells, CD57+ NK cells, CD68+ macrophages, CD19+ B cells, PD-1 protein, and cell nucleus (DAPI). Scale bar, 100 μM.

To elucidate improvement reasons of LIHC survival, we explored correlation of immune infiltration and FDX1 expression. Cibersort analysis indicated that immune infiltration in LIHC cases with low and high FDX1 expressions had a significant difference in proportion of T cells, macrophages, resting mast cells, and eosinophils cells (**Figure 3A**). ssGSEA result showed that activated B cells, CD56dim NK cells, macrophages, monocytes, NK cells, eosinophil/neutrophil, and type 1/17 T helper cell had a higher expression in the high-FDX1 group, and many types of T cells had a higher expression in the low-FDX1 group (**Figure 3B**). Furthermore, we found that expression of PD-1 was decreased in high-FDX1 group (**Figure 3C**). These results might explain better survival in LIHC with a high expression of FDX1.

To further demonstrate these results, we screened patients with LIHC with a low or high expression of FDX1 (**Figure 3D**). CD8+ T cells, CD57+ NK cells, CD68+ macrophages, CD19+ B cells, and PD-1 were stained in LIHC samples. As shown in **Figure 3E**, NK cells, macrophages, and B cells in infiltration of the tumor tissue from high FDX1 cases were significantly enhanced. However, PD-1 expression was higher in low FDX1 cases (**Figure 3E**). Thus, change of tumor immune microenvironment was one of the main reasons of survival difference in patients with LIHC with a low/high FDX1 expression.

### FDX1 promotes cuproptosis of tumor cells in patients with LIHC

**Figure 4:**
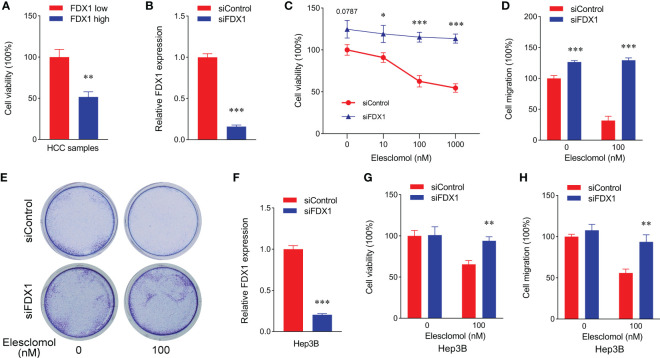
Low expression of FDX1 promoted proliferation and migration of hepatic cancer cells through decreasing cuproptosis. **(A)** Cell viability of HCC sample with low and high expressions of FDX1 (n = 5). Statistics based on the unpaired t-test. **p < 0.01. **(B)** FDX1 expression in siControl and siFDX1 HepG2 cells. Statistics based on the unpaired t-test. ***p < 0.001. **(C)** Cell viability of siRNA HepG2 cells treated with different dose of Elesclomol for 24 h (n = 5). Statistics based on the unpaired t-test. *p < 0.05 and ***p < 0.001. **(D)** Cell migration was detected in siRNA HepG2 cells after different treatments using cell viability analysis (n = 5). Statistics based on the unpaired t-test. ***p < 0.001. **(E)** Representative colony formation images of siRNA HepG2 cell staining with crystal violet staining after Elesclomol treatment using a Transwell coculture method. **(F)** FDX1 expression in siControl and siFDX1 Hep3B cells. Statistics based on the unpaired t-test. ***p < 0.001. **(G)** Cell viability of siRNA Hep3B cells treated with or without 100 nM Elesclomol for 24 h (n = 5). Statistics based on the unpaired t-test. **p < 0.01. **(H)** Cell migration was detected in siRNA Hep3B cells after different treatments using cell viability analysis (n = 5). Statistics based on the unpaired t-test. **p < 0.01.

FDX1 was demonstrated as a key regulatory factor of the cuproptosis pathway (9). Then whether cuproptosis also was one of the main reasons in survival improvement of LIHC with a high FDX1 expression, we detected viability of tumor cells from LIHC cases with a low/high FDX1 expression. As shown in **Figure 4A**, tumor cell viability in the low-FDX1 group was higher than that in the high-FDX1 group. The similar result was found in HepG2 cells with FDX1 knockdown (**Figures 4B, C**). Meanwhile, Cu^2+^ tolerance in HepG2 cells with FDX1 knockdown was significantly increased compared with the control group (**Figure 4C**), which suggested that tumor cells with a high FDX1 expression was easier to be caused cuproptosis at the same Cu^2+^ content. Migration of HepG2 cells with FDX1 knockdown was also markedly enhanced (**Figures 4D, E**). The consistent results were also found in Hep3B cells after knockdown of FDX1 expression (**Figures 4F–H**). These data revealed that a high expression of FDX1 promoted cuproptosis of LIHC cells to decrease prefiltration and migration abilities of tumor cells, which was also one of the main reasons in survival improvement of LIHC with a high FDX1 expression.

## Discussion

Cuproptosis involved in the improvement of survival in patients with HCC through the FDX1-mediated cell death pathway. Our study using the LIHC cohort of TCGA dataset and a 57-HCC retrospective cohort demonstrated that a high expression of FDX1 could enhance OS, PFI, DSS, and DFI of patients with HCC. High FDX1 expression or high Cu^2+^ concentration promoted cuproptosis of HCC cells, which was a reason for the improvement of survival in patients with HCC. Meanwhile, immune infiltration, such as NK cells, macrophages, and B cells, was higher and PD-1 expression was lower in the high–FDX1 expression group with HCC compared with that in the low–FDX1 expression group. These results showed that a high expression of FDX1 promoted immune cells to infiltrate HCC tissues and induced immune response. We speculated that the change of tumor immune microenvironment might be caused by cuproptosis-induced neoantigen production in patients with HCC with different expression of FDX1.

Copper homeostatic mechanisms were systematically reported by Tsvetkov’s team in 2022, which first revealed FDX1-mediated cuproptosis through the aggregation of lipoylated proteins and destabilization of Fe-S cluster proteins (9). Since then, the research studies of cuproptosis in tumors were of widespread interest. Many studies explored the effects of cuproptosis on different tumors, such as lung adenocarcinoma (LUAD) (13), kidney renal clear cell carcinoma (KIRC) (14), and LIHC (15), using the TCGA and/or Gene Expression Omnibus (GEO) databases. In LUAD, patients with a high risk score using the least absolute shrinkage and selection operator Cox regression algorithm had a worse survival (13). High cuproptosis scores caused poor prognosis in KIRC (14). The high expression of FDX1 exhibited a longer survival than the low-expression group in TCGA-LIHC, GSE14520, and the liver cancer project (code: LIRI_JP) of the International Cancer Genome Consortium (ICGC) (ICGC-LIRI) cohorts (15). Our study also found the similar result, which was demonstrated using a clinical cohort with 57 HCC cases. In LIHC cells, a high FDX1 expression or Cu^2+^ content promoted cell cuproptosis and decreased cell prefiltration and migration. Thus, cuproptosis was one of the main reasons in survival improvement of LIHC with a high FDX1 expression.

Tumor neoantigen-induced antitumor immunity is a favorable attention of tumor immunotherapy, except for immune checkpoint inhibitors (ICIs). Production of tumor neoantigen induced by pyroptosis, necrosis, and ferroptosis was demonstrated from more and more evidences. Radiotherapy was considered as turning immunologically “cold” tumors “hot” through cancer death-causing the release of pro-inflammatory mediators and neoantigen, which increased tumor immune cell infiltration (16–18). High-frequency irreversible electroporation produced the neoantigens through causing pyroptosis and necrosis of tumor cells *in vivo* and *in vitro* (19, 20). Ferroptosis induced tumor-specific immune responses and enhanced the effect of immunotherapy (21, 22). These research studies suggested that cuproptosis of tumor cells might also cause production of tumor neoantigens. In the present study, a high expression of FDX1 promoted death of LIHC cells, and patients with HCC with a high FDX1 expression displayed higher immune cell infiltration compared with the low FDX1 expression cases. Cuproptosis-induced neoantigen production might be involved in remodeling of tumor immune microenvironment and enhance survival of patients with HCC.

This study reveals reasons of survival improvement in patients with HCC with a high expression of FDX1 from two sides: Immune infiltration is high and PD-1 expression is low in the patients with HCC with a high expression of FDX1; a high FDX1 expression reduces tumor prefiltration and migration abilities through the cuproptosis pathway. Overall, cuproptosis and tumor immune microenvironment were together involved in improvement of survival in patients with HCC with a high expression of FDX1.

## Data availability statement

The original contributions presented in the study are included in the article/**Supplementary Material**. Further inquiries can be directed to the corresponding author.

## Ethics statement

The studies involving human participants were reviewed and approved by the Ethics Committee of Zhuhai People’s Hospital. The patients/participants provided their written informed consent to participate in this study. Written informed consent was obtained from the individual(s) for the publication of any potentially identifiable images or data included in this article.

## Author contributions

YQ and LC designed the research and supervised the study. YQ, WL and RY performed experiments. YQ, JC and HX analyzed the data. YQ wrote the paper, and LC revised the paper. All authors contributed to the article and approved the submitted version.
