# Supplementary material for: Tumor cuproptosis and immune infiltration improve survival of patients with hepatocellular carcinoma with a high expression of ferredoxin 1
Source: Front Oncol. 2023 Jun 8;13:1168769. doi: 10.3389/fonc.2023.1168769 (PMC10285401; doi:10.3389/fonc.2023.1168769)
Supplement: Supplementary file 1 [file Table_1.docx]

Supplementary materials


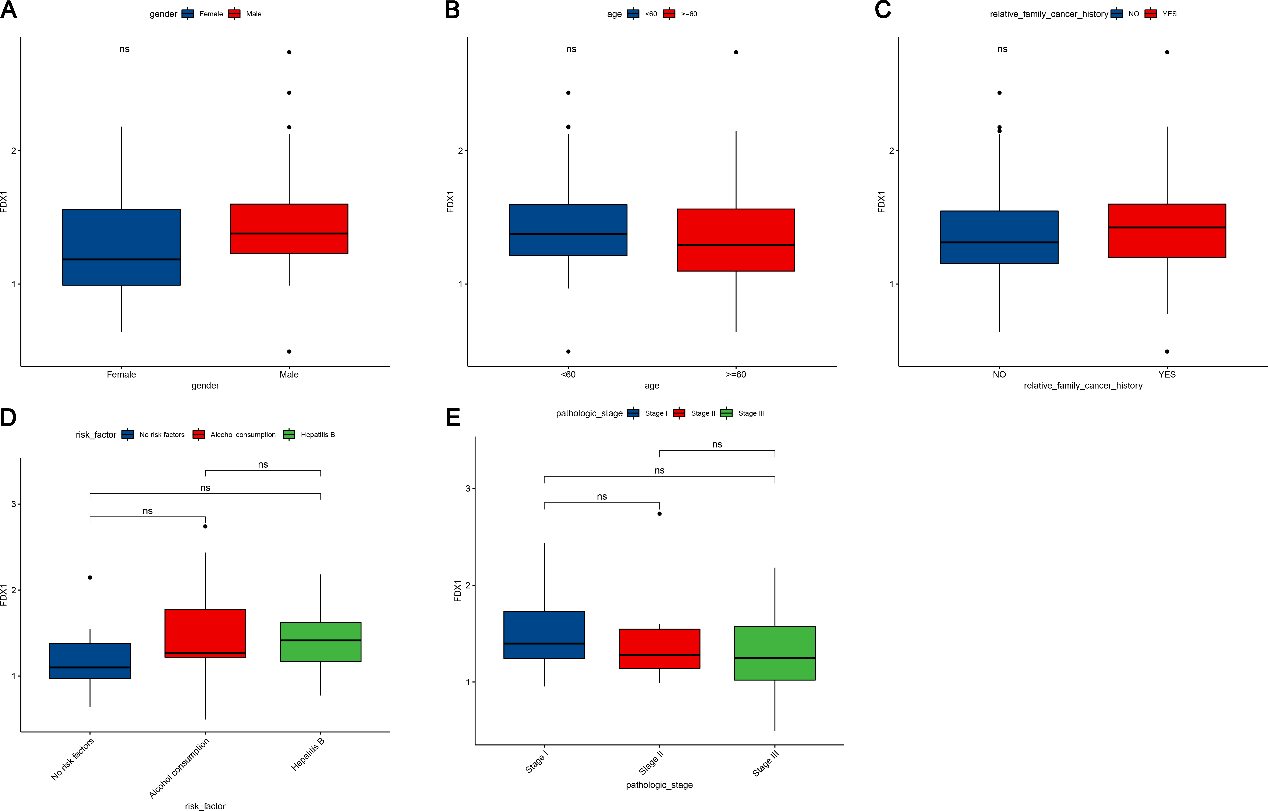


Figure S1. Effects of clinical characteristic on expression of FDX1 in HCC. (A-E) Correlation between FDX1 expression and gender (A), age (B), family cancer history (C), risk factor (D), pathologic stage (E) in HCC (n = 57). Statistics based on the two-tailed Mann-Whitney U test.
